# Age- and Activity-Related Differences in the Abundance of Myosin Essential and Regulatory Light Chains in Human Muscle

**DOI:** 10.3390/proteomes4020015

**Published:** 2016-04-08

**Authors:** James N. Cobley, Zulezwan Ab. Malik, James P. Morton, Graeme L. Close, Ben J. Edwards, Jatin G. Burniston

**Affiliations:** 1 Division of Sport and Exercise Science, Abertay University, 40 Bell Street, Kydd Building, Dundee DD1 1HG, UK; j.cobley@abertay.ac.uk; 2 Faculty of Sport Science and Coaching, Universiti Pendidikan Sultan Idris, Tanjung Malim, Perak 35900, Malaysia; zulezwan@fsskj.upsi.edu.my; 3 Research Institute for Sport and Exercise Sciences, Liverpool John Moores University, Liverpool L3 3AF, UK; j.p.morton@ljmu.ac.uk (J.P.M.); g.l.close@ljmu.ac.uk (G.L.C.); b.j.edwards@ljmu.ac.uk (B.J.E.)

**Keywords:** ageing, human skeletal muscle, myosin heavy chain, myosin light chain, selective reaction monitoring

## Abstract

Traditional methods for phenotyping skeletal muscle (e.g., immunohistochemistry) are labor-intensive and ill-suited to multixplex analysis, *i.e.*, assays must be performed in a series. Addressing these concerns represents a largely unmet research need but more comprehensive parallel analysis of myofibrillar proteins could advance knowledge regarding age- and activity-dependent changes in human muscle. We report a label-free, semi-automated and time efficient LC-MS proteomic workflow for phenotyping the myofibrillar proteome. Application of this workflow in old and young as well as trained and untrained human skeletal muscle yielded several novel observations that were subsequently verified by multiple reaction monitoring (MRM). We report novel data demonstrating that human ageing is associated with lesser myosin light chain 1 content and greater myosin light chain 3 content, consistent with an age-related reduction in type II muscle fibers. We also disambiguate conflicting data regarding myosin regulatory light chain, revealing that age-related changes in this protein more closely reflect physical activity status than ageing *per se*. This finding reinforces the need to control for physical activity levels when investigating the natural process of ageing. Taken together, our data confirm and extend knowledge regarding age- and activity-related phenotypes. In addition, the MRM transitions described here provide a methodological platform that can be fine-tuned to suite multiple research needs and thus advance myofibrillar phenotyping.

## 1. Introduction

Musculoskeletal ageing is characterized by age-related muscle fiber atrophy, as a corollary of motor unit (MU) loss [1]. Each MU comprises a presynaptic motor neuron and a postsynaptic population of mono-innervated skeletal muscle fibers [2]. MUs are, typically but not always [3], defined by uniform skeletal muscle fiber isoform (Type I, Type II) content [4]. Type II MUs are preferentially lost with age, owing to failed re-innervation [2]. Functionally, type II MU loss underpins age-related phenotypical alterations (e.g., reduced skeletal muscle power [5]), owing to the distinct contractile and metabolic properties associated with each fiber type (reviewed in [4,6]). Therefore, classification of skeletal muscle fiber type can provide insight into the nature of the phenotypical alterations that define musculoskeletal ageing [7].

Traditional biochemical and histochemical approaches to characterizing skeletal muscle phenotype involve assessment of metabolic enzyme activity (e.g., citrate synthase activity) and myosin heavy chain (MyHC) isoform content and/or ATPase activity [8,9]. Such approaches have revealed that musculoskeletal ageing is associated with reduced citric acid cycle and electron transport chain subunit activity and content [10,11], although this is modifiable by physical activity levels [1]. Ageing is also associated with reduced MyHCIIa and MyHCIIx expression, consistent with type II MU loss [7]. Unfortunately, the techniques used to collect the aforementioned data require relatively large amounts of sample and are ill-suited to multiplex analysis so typically just a few proteins are studied in isolation [9,12,13,14] and in a serial rather than parallel manner. The latter is a particular concern because metabolic and contractile differences can be uncoupled [12,15]. Resultantly, metabolic differences can exist despite similar MyHC isoform content [12]. The age-related appearance of hybrid skeletal muscle fibers that co-express multiple MyHC isoforms further complicates this issue [16]. The collective limitations of traditional biochemical and histochemical approaches preclude complete characterization of age and physical activity level phenotypes in skeletal muscle.

Proteomic approaches to characterizing skeletal muscle phenotype confer several important advantages, notably the capacity to analyze multiple proteins in a parallel and un-biased manner [17,18,19,20]. Consequently, proteomic research has advanced understanding of the phenotypical changes associated with musculoskeletal ageing [7,10,21,22,23,24,25]. For example, Gelfi *et al.* [7] reported myosin regulatory light chain (MLRS) content was lower in older compared with younger individuals, which may contribute to age-related contractile dysfunction [26]. Nevertheless, existing literature is broadly limited by two major caveats. First, the confounding influence of activity levels has seldom been standardized or controlled in human studies. For instance, lower MLRS isoform content of elderly individuals [7] could reflect reduced physical activity rather than a direct effect of ageing *per se*. Second, inconsistencies exist between proteomic studies in model species (e.g., rodents) compared with humans. For example, in contrast to humans, commonly used rodent models are inbred and rodents express a fast type MyHCII isoform (Type II b [9,15]), which is not present in human locomotive muscles.

Two-dimensional gel electrophoresis (2DGE) has been commonly employed in muscle proteomic investigations but this technique has widely acknowledged [12,19,21,27,28,29] technical limitations. In an effort to overcome some of the technical issues associated with 2DGE, we have used a high-performance liquid chromatography coupled mass spectrometry (LC-MS) that affords time-efficient (180 min per sample), automated and quantitative analysis of skeletal muscle samples [12]. Recent application of this approach identified heart-type fatty acid binding protein as a novel biomarker of aerobic capacity in both rodent and human skeletal muscle samples [12]; confirming the utility of this novel phenotypical approach. We reasoned that application of LC-MS to the myofibrillar fraction may advance phenotyping by enabling the measurement of isoform expression and splice variation of the myofibrillar proteome. Accordingly, the aims of the present study were to: (1) quantify differences in MyHC content using traditional denaturing gel electrophoresis methods; then (2) mine the myofibrillar proteome using LC-MS/MS; and (3) investigate differences between muscle samples of young and elderly adults using LC-MS label-free profiling. In addition, trained and untrained participants were included in each age group to further dissect the influence of physical activity levels on phenotypical biomarkers of ageing [10,11,30]. Finally, we used multiple reaction monitoring to verify discoveries generated by LC-MS profiling.

## 2. Results

### 2.1. MyHC Isoform Analysis

**Figure 1:**
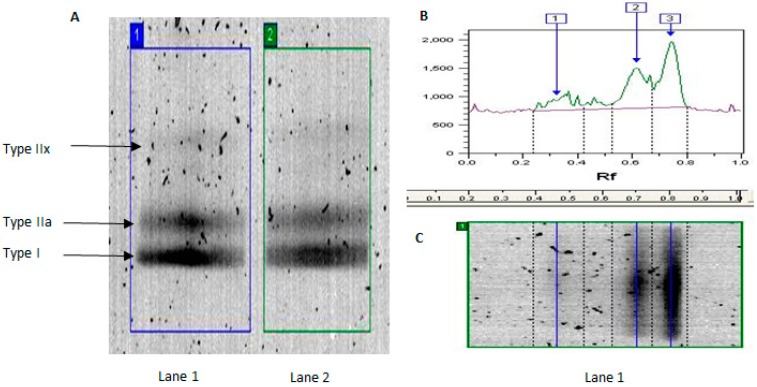
Separation of MyHC isoforms in human skeletal muscle using denaturing gel electrophoresis: (**A**) representative image of gel lanes showing separation of MyHC isoforms; (**B**) density plot showing area of interest, into distinct bands (IIx, IIa, I) based in differences in migration (pooled standard, *n* = 24); and (**C**) images of lanes aligned with the area of interest.

**Figure 2:**
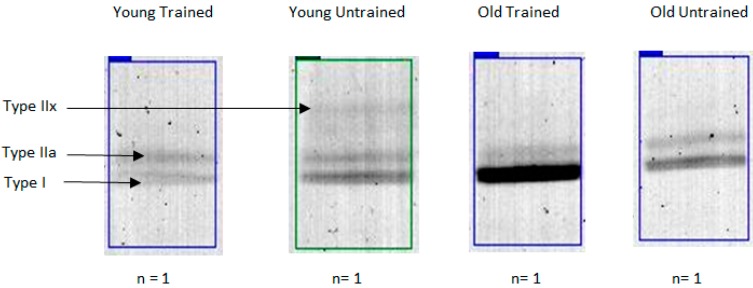
Influence of age and activity levels on MyHC isoform content in human skeletal muscle. Representative image of gel lanes from young trained (**YT**), young untrained (**YU**), old trained (**OT**) and old untrained (**OU**) individuals with clear separation of MyHC isoforms into distinct bands.

Denaturing gel electrophoresis conditions were optimized using a pooled standard (10 µg protein from each individual, *n* = 24). MyHC isoforms (MyHCI, MyHCIIa and MyHCIIx) were successfully resolved into three distinct bands (Figure 1). MyHC isoform content (MyHCI: 52.4% ± 1.7%, MyHCIIa: 38.4% ± 3.9% and MyHCIIx: 9.2% ± 2.2%) was consistent with previous literature [31]. Satisfied with the optimization of our electrophoresis protocol, we next analyzed individual samples (Figure 2). Endurance trained individuals typically exhibit greater Type I fiber content and thus lower Type IIa and Type IIx content compared with untrained individuals, as a function of genetic and environmental factors [3,6,15]. Consistent with this, percentage MyHCI and MyHCIIa levels were greater and lower, respectively, in trained compared with untrained participants (Table 1), irrespective of age [7]. Type IIa and Type IIx muscle fibers are preferentially lost with age as a consequence of failed re-innervation [2]. Accordingly, we report MyHCIIa levels were lower in older compared with younger participants, regardless of training status (Table 1). In addition, MyHCIIa and MyHCIIx levels were lower in the old trained (OT) compared with young trained (YT) group. Taken together, our MyHC isoform content analysis confirms known age- and physical activity-related phenotypes.

### 2.2. LC-MS-MS Mining of the Myofibrillar Proteome

**Figure 3:**
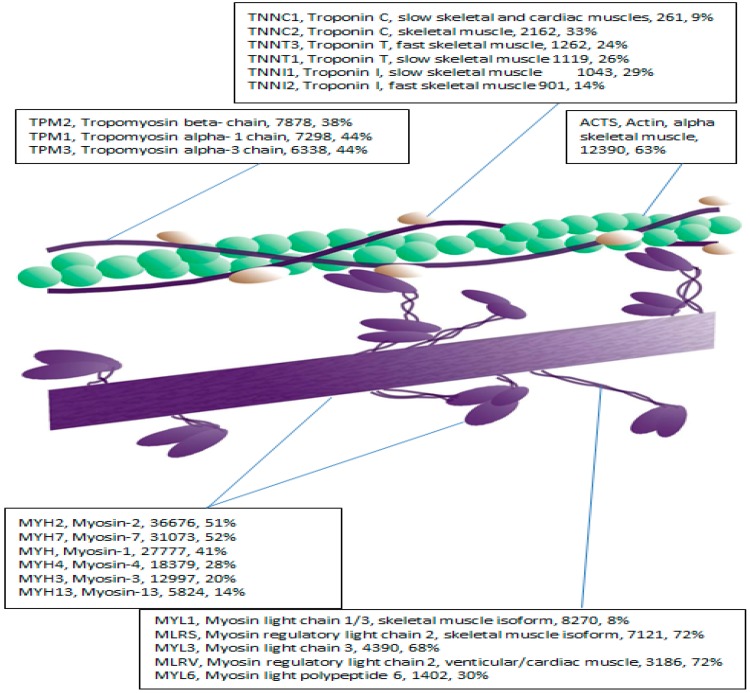
Illustration of the structure of thin filament (actin) and thick (myosin) filament; and its main protein components that are known to exist as multiple isoforms. Protein identifiers and descriptions are from the SwissProt database. The MOWSE score and sequence coverage for each protein are also reported from the database searches.

Denaturing gel electrophoresis-based MyHC isoform staining approaches are laborious and time consuming, *i.e.*, typically requiring electrophoresis at a stable temperature for ~15 h. Therefore, we investigated whether mass spectrometry-based analyses could provide a more comprehensive and time efficient analysis of muscle phenotype. We first mined the myofibrillar fraction (pooled standard) before quantifying phenotypical differences in individual samples. LC-MS/MS mining of the myofibrillar fraction of a pooled standard identified 47 proteins (M and SD: Mascot Score 5295 ± 8146; Sequence coverage: 34.6% ± 20.4%; Number of peptides: 10 ± 17; Table 2), which represents the most abundant myofibrillar proteins [28], including the actin-myosin complex, troponins, myosin light chains and tropomyosins (Figure 3). This demonstrates the potential utility of LC-MS profiling to identify and study multiple isoforms of common myofibrillar proteins in a semi-automated and time-efficient manner.

### 2.3. Differences in the Myofibrillar Proteome Due to Age or Habitual Levels of Physical Activity

Label-free LC-MS profiling matched 265 peptides across samples from young untrained (YU), young trained (YT), old untrained (OU) and old trained (OT) individuals. Statistical analysis of these data discovered 19 peptides from six proteins exhibited age- and physical activity-related differences (Table 3). Six peptides were specific for different isoforms of myosin essential or regulatory light chains, whereas peptides belonging to MyHC could not be used to unambiguously identify specific isoforms.

Peptide data from label-free profiling were analyzed by two-way analysis of variance. Data represent statistical output (*p* values) of the main comparisons for age (young *versus* old) or training status (trained *versus* untrained) or the interaction between age and training status for each peptide. The retention time and mass-to-charge ratio of each peptide is reported and proteins were identified from the SwissProt database: skeletal muscle actin (ACTS), skeletal muscle myosin regulatory light chain (MLRS), myosin heavy chain (MYH), myosin essential light chain 1 (MYL1), myosin essential light chain 3 (MYL3) and tropomyosin (TPM).

### 2.4. Confirmation of Age- and Activity-Related Differences in Myosin Light and Regulatory Chain Isoforms Using Selective Reaction Monitoring

**Figure 4:**
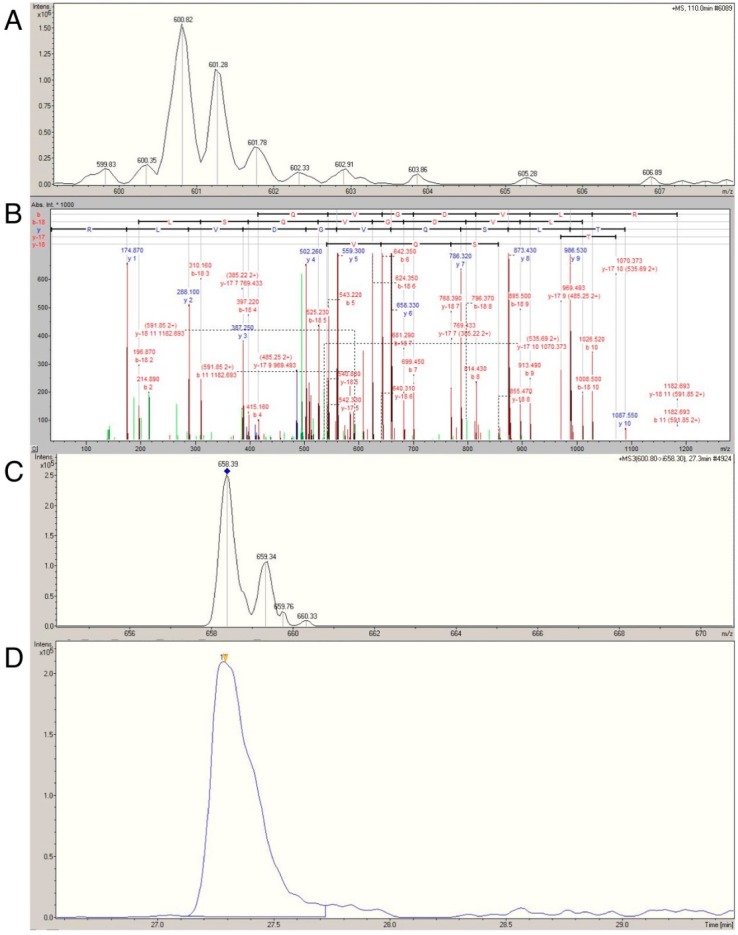
Selective reaction monitoring of MYL1 transition 600.8 → 658.3. Parent ion MS spectra (**A**) and annotated MS/MS spectra (**B**) of the doubly-charged peptide ITLSQVGDVLR spanning residues 70–80 of MYL1_HUMAN. Selective monitoring (**C**) of the y6 ion (*m/z* 658.3) was used and relative abundance was measured as area under the curve of the extracted ion (*m/z* 600.8 → 658.3) chromatogram (**D**).

Label-free LC-MS profiling of the human myofibrillar proteome discovered MLRS, MYL1 and MYL3 abundance may significantly differ as a function of age and physical activity. To verify these discoveries, we further performed selective reaction monitoring of isoform specific tryptic peptides [12]. The utility of this approach was first confirmed by programming the mass spectrometer to selectively monitor single isoform-specific transitions, for example the doubly charged precursor ion (600.82 *m*/*z*) corresponding to ITLSQVDVLR of human MYL1 and the selective transition to the y6 Fragment ion (658.3 *m*/*z*) illustrated in Figure 4. Thereafter, we used multiple reaction monitoring (MRM) to quantify (Table 4) the following isoform-specific transitions: 505.7 → 550.2 (MYL1), 600.8 → 658.3 (MYL1), 771.7 → 835.4 (MYL1), 498.2 → 759.4 (MYL3), 600.2 → 735.4 (MLRS) and 762.8 → 780.2 (MLRS).

**MYL1:** MRM analysis of MYL1 revealed that each isoform specific transition was significantly (*p* ≤ 0.05) greater in young compared with old participants, irrespective of training status (Table 4). Accordingly, mean MYL1 isoform abundance was significantly greater (*p* ≤ 0.05) in young compared with old participants. MYL1, is principally expressed in fast twitch fibers [32] and MYL1 abundance was significantly less in OT (*p* = 0.004) and OU (*p* = 0.045) groups compared with YU group (Table 5). There was also a trend (*p* = 0.63) towards greater MY1 abundance in YU compared with YT. Altogether, MYL1 data are consistent with greater fast twitch fiber abundance in trained compared with untrained participants and an age-related loss of fast twitch muscle fibers in skeletal muscle [6,7,15,21].

**MYL3:** MYL3, which is enriched in slow skeletal muscle fibers [13,33], displayed the reverse profile being significantly (*p* = 0.021) greater in older compared with younger participants, with a trend (*p* = 0.083) towards higher levels in trained compared with untrained participants (Table 4). MYL3 abundance was significantly (*p* = 0.038) less in YU compared with OT. MYL3 abundance was not different (*p* ≥ 0.05) between YT and OT groups (Table 5). MYL3 data are, therefore, consistent with the greater type I fiber content in older compared with younger skeletal muscle [6,7,15,21], driven by lower levels in YU compared with OT.

**MLRS:** Mean MLRS (also referred to MLC2-f) content was significantly (*p* = 0.031) greater in untrained compared with trained participants, with a trend (*p* = 0.055) towards lower MLRS content in older compared with younger participants (Table 4). Mean MLRS abundance (Table 5) was significantly (*p* = 0.030) greater in YU compared with OT, whereas mean MLRS abundance did not differ (*p* ≥ 0.05) between YU and YT. Therefore, our MLRS data are broadly consistent with greater fast twitch fiber content in untrained compared with trained participants [6,7,15,21].

Collectively, our data demonstrate that isoform specific myosin light chain transitions are useful phenotypical biomarkers of age and activity related phenomena in human skeletal muscle.

## 3. Discussion

We used traditional biochemical analysis of MyHC isoforms to confirm age-related differences in human muscle phenotype and further demonstrate the effect of habitual activity on these data. We expand on our previous work [12,28], by using label-free LC-MS profiling to phenotype the human myofibrillar proteome. We resolved 47 myofibrillar proteins without prior orthogonal separation, which is consistent with the well-documented high-abundance of a small number of contractile proteins in skeletal muscle [17,19,20]. Indeed, just ten contractile and metabolic proteins account for over half of the total protein content in skeletal muscle [29,30]. LC-MS profiling detected significant differences in the abundance of peptides belonging to essential and regulatory myosin light chains between young and elderly individuals that were either sedentary or undertook regular exercise training. To verify these findings, we performed MRM of selected isoform-specific peptides using a condensed LC gradient with a sample turn-around of 40 min. In addition to verifying novel information regarding age-related differences in myosin light chain abundances, future researchers are able to capitalize on these findings and also perform rapid, semi-automated and parallel profiling of muscle proteins using our reported transitions.

Consistent with previous literature [7,21,31], MyHCIIa abundance, a marker of Type IIa fiber content, was less in older compared with younger individuals, regardless of training status. Indeed, MyHCIIx, a marker of Type IIx content, was almost completely absent in the OT group. MyHCIIa and MyHC IIx phenotypes are likely to be associated with the age-related preferential loss of Type II MU [2,34]. The molecular sequelae underpinning Type II MU loss are unclear but this phenomenon may be driven by aberrant repulsive axonal guidance (e.g., Semaphorin 3A) mediated denervation [34,35,36]. Irrespective of age, trained participants displayed greater MyHCI and lesser MyHCIIa abundance compared with untrained participants. This is consistent with greater Type I and lesser Type IIa fiber content in endurance-trained individuals owing to genetic and environmental factors [6,15]. Whilst our cross-sectional design precludes causative analysis [10], MyHC data are consistent with known age- and activity-related phenotypes [7,21,25]. Our ability to confirm existing paradigms of age- and activity-related MyHC phenotypes using traditional analysis meant we could largely exclude the possibility that discoveries made by LC-MS profiling were entirely specific to the population of individuals and instead can be discussed alongside existing literature.

Label-free profiling overcomes many of the technical limitations associated with 1D and 2D gel electrophoresis and could significantly advance phenotyping of skeletal muscle. Nonetheless, LC-MS analysis based solely on intensities of parent ion masses can be confounded by interference from unrelated peptides that share analogous retention times and mass-to-charge ratios [12]. With this limitation in mind, we sought to verify the age- and activity-related differences in actin, myosin, tropomyosin, MLRS, MYL1 and MYL3 using multiple reaction monitoring (MRM; reviewed in [37]) This technique selectively monitors and quantifies the intensity of daughter ions from known parent ions (termed transitions [12]), which greatly enhances the specificity of the analysis. We previously used MRM to confirm that heart-type fatty acid protein was more abundant in rodents artificially selected for higher aerobic capacity compared with lower aerobic capacity [12], affirming the utility of this approach in skeletal muscle. Application of MRM to the myofibrillar fraction presented a significant analytical challenge owing to high myofibrillar protein sequence homology. For example, MYL1 and MYL3 share 72.6% sequence identify. Consequently, only isoform specific transitions in selected proteins were analyzed.

Myosin essential (MYL1, MYL3) and regulatory (MLRS) light chains were selected for MRM analysis owing to their essential role in regulating contractile velocity [38] and prior reports of differential expression in old compared with young skeletal muscle [7,22]. MYL1 and MLRS are predominately, but not exclusively [39,40], expressed in fast twitch muscle, whereas MYL3 is predominately expressed in slow twitch muscle [13,24]. Using known age and activity related phenotypes as a conceptual framework [2,3,6,21,34], it could be predicted, *a priori*, that: (1) MYL1 and MLRS content should be greater; and (2) MYL3 content should be lesser in the skeletal muscle of elderly compared to young adults. The prediction for MLRS is complicated by conflicting reports of an age-related decrease in MLRS in humans [7] against a drastic increase in MLRS that demarcates the onset of senescence in rodent skeletal muscle [26]. Whilst this could be related to interspecies differences [41], it could equally be attributable to a failure to control for physical activity status particularly in human studies [1,42]. We reasoned that the latter was more likely owing to a recent report documenting lower MRLS content in trained compared with recreational runners [43]. In support of this, we report novel evidence that age-related changes in MLRS abundance could easily be confounded by the physical activity status of the participants. Specifically, MLRS differed as a function of training status and the trend towards differential age-related expression was owing to significantly greater levels in YU compared with OT, but not YT compared with OT. This observation warrants further mechanistic exploration. Future reports may wish to extend our observations by comparing the relative abundance and phosphorylation status of each of the fast and slow regulatory isoforms.

We also report novel data identifying that MYL1 and MYL3 content is lesser and greater, respectively, in the muscle of old compared with younger adults. As a consequence, the ratio between MYL3 and MYL1 is dramatically different between young, elderly, trained and untrained humans (Table 4 and Table 5). Our findings are the first of their kind rendering comparison with previous literature difficult. This notwithstanding, MYL1, MYL3 and MLRS data confirm our MyHC analysis and are consistent with the notion that ageing is accompanied by preferential Type II MU loss [2,34]. It will, however, be important to confirm this observation with longitudinal studies that permit greater mechanistic insight; as one cannot rule out the possibility that innate fiber type differences between our groups underpinned our observations and not age or physical activity status *per se*. Such caveats should be viewed globally; that is, they are generic to the research field, being unrestricted to the present study owing to the difficulties associated with conducting longitudinal studies of human ageing.

MRM affords semi-automated, rapid and quantitative phenotyping [12,37] and the potential wider application of this approach has not escaped our attention. Future investigators may wish to use our data as a resource to perform isoform specific MRM analysis of myosin light chain content; thus, obviating the limitations of traditional methods of muscle phenotyping. In the future, this approach could be further extended by spiking samples with stable isotope-labeled peptides to provide quantitative analysis of human samples.

## 4. Materials and Methods

### 4.1. Participants

Ethical approval was granted from the Liverpool John Moores University Ethics Committee and the present study adhered to the declaration of Helsinki. After providing written informed consent, 12 younger (18–30 years) and 12 older (≥55 years) Caucasian males participated and were segregated according to training history to yield four experimental groups: young trained (YT, *n* = 6), young untrained (YU, *n* = 6), old trained (OT, *n* = 6) and old untrained (OU, *n* = 6). Trained participants were competitive amateur cyclists that had habitually completed at least five endurance sessions per week. Untrained participants did not participate in systematic endurance training and completed ≥3 exercise sessions per week. Basic participant anthropometrical and physiological characteristics have been reported previously [42,44].

### 4.2. Muscle Biopsies

After the administration of a local anesthetic (0.5% Marcaine), muscle biopsies were obtained from the *Vastus Lateralis* using a Bard Monopty disposable biopsy instrument (12 cm × 10 cm gauge, Bard Monopty Systems, Tempe, AZ, USA). Muscle samples were immediately snap frozen in liquid nitrogen and stored at −80 °C until further analysis.

### 4.3. MyHC Isoform Staining

Approximately 20–30 mg of frozen muscle tissue was ground to powder and homogenized in 120 μL of ice cold lysis buffer supplemented with phosphatase and protease inhibitors (25 mM Tris/HCl (pH 7.4), 50 mM NaF, 100 mM NaCl, 5 mM EGTA, 1 mM EDTA, 10 mM Na-Pyrophosphatase, 1 mM Na_3_VO_4_, 0.27 M sucrose, 1% Triton X-100, 0.1% 2-mercaptoethanol). Homogenates were centrifuged at 1000 *g* for 10 min at 4 °C. The resultant pellet (myofibrillar protein) was re-suspended in 1% SDS buffer and boiled at 95 °C for 5 min. Protein content was determined using a Bradford assay [45]. Samples (10 µL equivalent to 0.5 µg·µL^−1^ protein) were separated by molecular weight using an 8% denaturing gel electrophoresis protocol (see [29,30]). Samples were electrophoresed on ice for 15 h at 280 V. Polyacrylamide gels were washed (ddH_2_O) and stained with Coomassie blue, as previously described [46,47]. Bands were quantified by densitometry analysis on T120 (Total lab., Newcastle upon Tyne, UK) software.

### 4.4. Label Free Liquid Chromatography Coupled Mass Spectrometry (LC-MS)

The myofibrillar faction was prepared for in-solution digest by precipitating supernatants in 5 volumes of acetone, re-suspending in 0.1% (*w*/*v*) Rapigest SF (Waters; Milford, MA, USA) and 50 mM ammonium bicarbonate before incubation at 80 °C for 15 min. Pellets were reduced with DTT (final concentration 1 mM) at 60 °C for 15 min in the dark before being alkylated with 5 mM at 4 °C for 30 min. Sequence grade trypsin (Promega, Madison, WI, USA) was added at a protein ratio of 1:50 and digestion allowed to proceed at 37 °C overnight. Digestion was terminated by 2 μL concentrated TFA addition. Tryptic peptide solutions were cleared by centrifugation at 13,000 *g* for 15 min at room temperature.

Label free LC-MS analysis was performed on a quadrupole-high capacity ion-trap (HCT Ultra ETD II; Bruker Daltonics, Bremen, Germany) coupled to an online electrospray ionization and nano-flow HPLC system (Ultimate 3000; Dionex, Sunnyvale, CA, USA). Consistent with previous work [12], tryptic digests (0.8 μg/μL) were diluted 1:10 in 0.1% formic acid (FA) solution. Samples (5 µL) were loaded onto a pre-column (Agilent Technologies Ltd., Santa Carla, CA, USA) and separated on Zorbax 300SB C_18_ 3.5 μm, 15 cm × 75 μm analytical reverse phase column (Agilent Technologies Ltd.) at a flow rate of 300 nL·min^−1^ using a non-linear gradient rising to 40% acetonitrile 0.1% FA over 160 min. Mass spectra for LC-MS profiling were recorded between 200 *m*/*z* and 2500 *m*/*z* using standard enhanced mode (8100 (*m*/*z*)/s). Equivalent data-dependent tandem mass spectrometry (MS/MS) spectra were collected from triplicate analysis of a pooled standard. MS/MS spectra of collision-induced dissociation fragment ions were recorded for the 5 most abundant precursors from each survey scan (350 to 1600 *m*/*z*).

Profile analysis (defined by two attributes: age and physical activity status) was used to calculate peptide regulation ratios. Peptides that significantly differed between group attributes were determined (Profile Analysis Version 4.0; Bruker Daltonics, Bremen, Germany). LC-MS/MS data were combined to identify proteins with a Swiss-Prot database restricted to “Human” on a locally implemented Mascot (www.matrixscience.com) server (version 2.2.03). Peptide/protein identifications were linked to the peptide regulation ratios determined in Profile Analysis and protein regulation ratios were calculated from peptide regulation ratios.

### 4.5. Selective Reaction Monitoring (SRM)

SRM [37] was used to verify and quantify differences in myosin light chain 1 (MYL1), myosin light chain 3 (MYL3) and myosin regulatory light chain 2 (MLRS) isoform specific tryptic digests and their associated precursor and product fragment ions. Samples were separated using a linear chromatographic gradient rising to 40% acetonitrile and 0.1% FA over 15 min. Samples were analyzed in duplicate and in a randomized order. Transitions of *m*/*z* 498–759 were monitored for MYL3, *m*/*z* 771–835 and *m*/*z* 600–658 for MYL1, *m*/*z* 660–735 and *m*/*z* 762–780 for MLRS.

### 4.6. Statistical Analysis

A two-way ANOVA was employed to determine the influence of age (*i.e.*, young *vs.* old) and physical activity (*i.e.*, trained *vs.* untrained) levels on outcome variables (e.g., MyHC content). To determine where significant differences occurred, significant main effects of age and activity levels were further explored with *post-hoc* Bonferroni tests. An α value of *p* ≤ 0.05 was used for all tests. Statistical analysis was performed on the Statistical Package for Social Sciences (SPSS, version 17.0, SPSS Inc.: Chicago, IL, USA). Data are presented as Mean and SD (±).

## 5. Conclusions

We used an advanced analytical workflow to perform phenotypic profiling of the human myofibrillar proteome. In doing so we discovered and verified novel differences in the abundance of essential myosin light chains MYL1 and MYL3 associated with age. Moreover, by studying well-defined populations of physically-active and sedentary individuals we were able to resolve previously conflicting data regarding differences in regulatory myosin light chain content in the muscle of young and elderly adults.

## Figures and Tables

**Table 1:** Quantitative analysis of MyHC isoform content by group.

Band	YT	YU	OT	OU	Effect of Age in Trained	Effect of Age in Untrained
IIx (%)	9.0 ± 8.3	13.8 ± 13.5	0.2 ± 0.4	9.5 ± 13.1	98% lower	31% lower
Iia (%)	35.6 ± 14.2	50.2 ± 15.1	21.7 ± 13.8 *	30.0 ± 20.7	40% lower	40% lower
I (%)	55.4 ± 27.8	36.0 ± 12.1	78.1 ± 14.2 *	60.5 ± 30.1	41% higher	68% higher

* Denotes significant difference (*p* ≤ 0.05) from YU.

**Table 2:** List of myofibrillar proteins identified by LC-MS-MS in a pooled standard.

Accession	Protein Name	Score	SC (%)	Peptides	MW	pI
MYH3	Myosin-3	12,997	20	1	223.8	5.5
MYH13	Myosin-13	5824	14	1	223.5	5.4
MYH7	Myosin-7	31,073	52	71	223.0	5.5
MYH1	Myosin-1	27,777	41	11	223.0	5.5
MYH2	Myosin-2	36,676	51	94	222.9	5.5
MYH4	Myosin-4	18,379	28	1	222.9	5.6
MYOM2	Myomesin-2	116	9	5	164.8	5.8
MYPC1	Myosin-binding protein C, slow type	763	17	14	128.2	5.7
ACTN2	Alpha-actinin-2	5705	45	31	103.8	5.2
LDB3	LIM domain-binding protein 3	1259	18	9	77.1	9.4
ALBU	Serum albumin precursor	1689	41	20	69.3	5.9
K2C1	Keratin, type II cytoskeletal 1	171	10	6	66.0	8.8
PDLI5	PDZ and LIM domain protein 5	135	15	4	63.9	9.6
K1C10	Keratin, type I cytoskeletal	116	6	3	58.8	5.0
ATPB	ATP synthase subunit beta, mitochondrial	261	21	6	56.5	5.1
DESM	Desmin	1778	39	15	53.5	5.1
VIME	Vimentin	424	14	2	52.6	4.9
ENOB	Beta-enolase	324	47	4	46.9	8.6
CASQ1	Calsequestrin-1	1718	11	2	44.5	3.9
KCRM	Creatine kinase M-type	5182	43	1	43.1	6.9
ACTS	Actin, alpha skeletal muscle	12,390	63	23	42.0	5.1
ACTB	Actin, cytoplasmic 1	6353	30	1	41.7	5.2
ALDOA	Fructose-bisphosphate aldolase A	1159	41.8	9	39.4	9.2
ALDOC	Fructose-biphosphate aldolase C	102	18	1	39.4	6.5
FHL1	Four and a half LIM domains protein 1	398	14	3	36.2	10.5
G3P	Glyceraldehyde-3-phosphate dehydrogenase	599	38	7	36.0	9.3
TNNT1	Troponin T, slow skeletal muscle	1119	26	6	32.9	5.8
TPM2	Tropomyosin beta- chain	7878	38	13	32.8	4.5
TPM3	Tropomyosin alpha-3 chain	6338	44	7	32.8	4.5
TPM1	Tropomyosin alpha- 1 chain	7298	44	11	32.7	4.5
TNNT3	Troponin T, fast skeletal muscle	1262	24	6	31.8	5.6
MYOZ1	Myozenin-1	599	51	7	31.7	9.3
CAH3	Carbonic anhydrase 3	760	38	6	29.5	7.1
MYL6B	Myosin light chain 6B	152	15	1	22.7	5.5
MYL3	Myosin light chain 3	4390	68	10	21.9	4.9
TNNI1	Troponin I, slow skeletal muscle	1043	29	5	21.7	10.3
TNNI2	Troponin I, fast skeletal muscle	901	14	3	21.3	9.6
MYL1	Myosin light chain 1/3, skeletal muscle	8270	8	15	21.1	4.8
MLRS	Myosin regulatory light chain 2, skeletal muscle	7121	72	2	19	4.7
MLRV	Myosin regulatory light chain 2, venticular/cardiac	3186	72	11	18.8	4.7
TNNC1	Troponin C, slow skeletal and cardiac	261	9	2	18.4	3.9
TNNC2	Troponin C, skeletal muscle	2162	33	4	18.1	3.9
MYG	Myoglobin	3163	55	8	17.2	7.9
MYL6	Myosin light polypeptide 6	1402	30	2	16.9	4.4
HBB	Hemoglobin subunit beta	11,403	88	12	16	6.9
HBD	Hemoglobin subunit delta	4206	69	2	16	9.1
HBA	Hemoglobin subunit alpha	2586	53	6	15.2	9.4

**Table 3:** Label-free LC-MS-MS profiling of the myofibrillar faction of individual samples.

Accession	Retention Time: Mass/Charge Ratio	*p*-Value			
		Age	Training	Age × Training	Sequence
ACTS	40.1 min: 644.31 *m*/*z*	0.552	0.289	0.02	GILTLK
	98.9 min: 895.86 *m/z*	0.024	0.315	0.336	SYELPDGQVITIGNER
	44.7 min: 1162.47 *m/z*	0.043	0.537	0.678	EITALAPSTMK
MLRS	83.4 min: 652.64 *m/z*	0.159	0.029	0.05	VAPEEHPTLLTEAPLNPK
	103.8 min: 1524.61 *m/z*	0.117	0.03	0.2	FLEELLTTQCDR
	32.1 min: 1173.43 *m/z*	0.06	0.026	0.399	DGIIDKEDLR
MYH	104.4 min: 1319.51 *m/z*	0.045	0.046	0.465	GADPEDVITGAFK
	117.6 min: 895.91 *m/z*	0.026	0.096	0.211	NAYEESLDQLETLK
	21.9 min: 1004.37 *m*/*z*	0.268	0.348	0.041	ELEEISER
	144.6 min: 1893.88 *m/z*	0.004	0.051	0.051	LLSTLFANYAGADAPIEK
	67.1 min: 1507.59 *m/z*	0.015	0.275	0.302	LTYTQQLEDLKR
	21.5 min: 1272.36 *m/z*	0.015	0.51	0.87	TLEDQMNEHR
MYL1	20.0 min: 1411.48 *m/z*	0.876	0.832	0.027	ELTYQTEEDRK
	17.8 min: 1482.51 *m/z*	0.108	0.555	0.033	KVQHELDEAEER
	119.4 min: 1010.42 *m/z*	0.508	0.022	0.444	EAFLLFDR
MYL3	116.4 min: 1542.60 *m/z*	0.001	0.841	0.798	DQATYEDFVEGLR
	22.9 min: 1524.60 *m/z*	0.03	0.917	0.662	AAPAPAPPPEPERPK
TPM	68.8 min: 1243.50 *m/z*	0.04	0.574	0.63	IQLVEEELDR

**Table 4:** Verification of myosin light chain abundances by multiple reaction monitoring.

Transition	Young	Old	Age (*p* Value)	Trained	Untrained	Activity (*p*-Value)
MYL1 (771.7 → 835.4)	1372 ± 669	796 ± 280	0.06	868 ± 388	1343 ± 684	0.024
MYL1 (600.8 → 658.3)	2790 ± 1309	1682 ± 640	0.007	1761 ± 848	2806 ± 1252	0.013
MYL1 (505.7 → 550.2)	3829 ± 2133	1764 ± 1187	0.007	2014 ± 1515	3744 ± 2149	0.025
MYL1 total	2081 ± 977	1239 ± 450	0.006	1315 ± 614	2075 ± 953	0.014
MLY3 (498.2 → 759.4)	3579 ± 1682	6353 ± 3347	0.021	5881 ± 3140	3867 ± 2403	0.083
MYL3:MYL1 ratio	1.72	5.13		4.47	1.86	
MLRS (660.2 → 735.4)	1029 ± 436	740 ± 399	0.108	701 ± 379	1097 ± 410	0.029
MLRS (762.8 → 780.2)	2101 ± 1135	1236 ± 834	0.048	1251 ± 904	2164 ± 1080	0.039
MLRS total	1565 ± 778	988 ± 591	0.055	976 ± 630	1630 ± 725	0.031

Data are presented as mean ± standard deviation (SD) of the area under the curve for each isoform specific transition. Main effect *p* values are presented for statistical analysis by age (young and old) and activity (trained and untrained) level.

**Table 5:** MRM transition area under the curve by group.

Isform Specific Transition	YT	YU	OT	OU
MYL1 (771.7 → 835.4)	1034 ± 482	1779 ± 672	703 ± 181 *	907 ± 356 *
MYL1 (600.8 → 658.3)	5777 ± 2532	5707 ± 7583	5457 ± 5430 *	8436 ± 8908
MYL1 (505.7 → 550.2)	2829 ± 1822	4829 ± 2072	1199 ± 373 *	2441 ± 1515
MYL1 Average	1568 ± 801	2698 ± 459	1062 ± 186 *	1451 ± 600 *
MYL3 (498.2 → 759.4)	4529 ± 1478	2438 ± 1166	7233 ± 3889 *	5296 ± 2556
*MYL3:MYL1 ratio*	*2.89*	*0.90*	*6.81*	*3.65*
MLRS (762.8 → 780.2)	1632 ± 116	2571 ± 980	869 ± 321 *	1675 ± 1081
MLRS (660.2 → 735.4)	829 ± 450	1229 ± 345	573 ± 273 *	939 ± 46
MLRS Average	1231 ± 794	1900 ± 658	721 ± 295 *	1307 ± 730

Data are presented as mean ± standard deviation (SD) of the area under the curve for each isoform specific transition. * Denotes significant difference (*p* ≤ 0.05) from YU group.

## Abbreviations

The following abbreviations are used in this manuscript:

2DGE	Two-dimensional gel electrophoresis
LC-MS	liquid chromatography mass spectrometry
MLRS	Myosin Light Regulatory Chain, fast isoform
MRM	Multiple Reaction Monitoring
MyHC	Myosin Heavy Chain
MYL1	Myosin Light Chain 1
MYL3	Myosin Light Chain 3
MU	Motor unit
OT	Old Trained
OU	Old Untrained
SRM	Selective Reaction Monitoring
YT	Young Trained
YU	Young Untrained
